# A Novel Silicon/Phosphorus *Co*-Flame Retardant Polymer Electrolyte for High-Safety All-Solid-State Lithium Ion Batteries

**DOI:** 10.3390/polym12122937

**Published:** 2020-12-09

**Authors:** Li Zeng, Lu Jia, Xingang Liu, Chuhong Zhang

**Affiliations:** State Key Laboratory of Polymer Materials Engineering, Polymer Research Institute of Sichuan University, Chengdu 610065, China; zengli_0718@foxmail.com (L.Z.); jialu_scu@163.com (L.J.)

**Keywords:** polymer electrolyte, silicon/phosphorus co-flame retardant, all-solid-state, lithium-ion battery

## Abstract

Developing a solid polymer electrolyte with superior flame retardancy and lithium-ion transportation properties is still a challenge. Herein, an intrinsic silicon/phosphorus co-flame retardant polymer solid electrolyte was prepared by using polyethylene glycol (PEG) co-polymerized with silicon and phosphorus-containing monomers. Due to the synergistic flame-retardant effect of silicon and phosphorus elements, this polymer electrolyte exhibits excellent thermal stability and flame resistance. Moreover, the ionic conductivity of the electrolyte at 25 °C is as high as 2.98 × 10^−5^ S/cm when the mass ratio of LiN(SO_2_CF_3_)_2_ (LiTFSI) and the prepared polymer electrolyte is 10:1. What is more, the LiFePO_4_/Li all-solid-state battery assembled with this solid electrolyte can work stably at a high temperature of 60 °C and exhibits a specific capacity of 129.2 mAh/g at 0.2 C after 100 cycles, providing a promising application prospect for high-safety lithium-ion batteries.

## 1. Introduction

Lithium-ion batteries (LIBs) are widely used in 3C electronics, energy storage systems and electric vehicles because of their high specific energy, high energy density and long lifetime cycle [1,2,3]. Electrode, electrolyte and the battery management system are the three important roles, and the normal operation of the devices needs the perfect cooperation of these three parts [4,5]. In recent years, safety problems—such as the burning and explosion of electric vehicles, laptops and cell phones caused by LIBs—have become increasingly prominent, and this restricts the practical application of LIBs. As the use of flammable liquid organic electrolytes in LIBs is the major cause of safety problems, the solid electrolyte interface (SEI) film between the liquid electrolyte and the negative electrode will be destroyed under conditions such as high temperature and pressure. The lithium metal reacts with the organic solvent of the electrolyte and releases flammable gases [6,7,8]. All these chemical reactions produce a lot of heat, which may cause thermal runaway and even safety accidents such as smoke, fire and explosion [9,10,11,12]. Therefore, using solid electrolytes without organic solvents can effectively solve the safety problem of LIBs. In particular, polymer solid electrolytes have the advantages of light weight, good flexibility, simple preparation process, and controllable size, all of which greatly improve the safety performance of LIBs and are conducive to improving the energy density of batteries [13,14,15,16]. However, polymer solid electrolytes still have some problems, such as low ionic conductivity at room temperature and poor safety [17].

Among all polymer electrolyte substrates, polyethylene oxide (PEO) is the most studied polymer matrix [18], which contains polar ethoxy groups in the molecular chain and can complex well with lithium salt and conduct lithium ions. However, shortcomings such as low room temperature conductivity and flammability have hindered the development of PEO-based electrolytes. In order to improve the safety performance of polymer electrolytes, current strategies include adding fire retardant fillers [19], chemical crosslinking [20] or grafting [21], and adding room temperature ionic liquids [22,23] or superionic conducting ceramics [24]. Chemical grafting stands out not only for its effective inhibition of PEO crystallization, which is beneficial to improve the ionic conductivity, but it also provides an intrinsic flame retardant polymer electrolyte matrix with excellent flame retardant performance [25]. Organosilicon compounds are some of the most typical flame retardants; they can form a silicon dioxide protective layer on the surface of polymer matrix during combustion to improve the flame resistance of a polymer matrix [26]. Silicon-containing polymers also possess good thermal stability and low heat combustion; however, poor ionic conductivity limits their application in lithium-ion batteries. On the other hand, phosphates are a kind of flame retardant, with a long history, that can produce phosphate radicals to capture active oxygen radicals and promote the carbonization of the matrix and prevent further combustion. Meanwhile, phosphates can also be used as electrolytes: phosphorus and silicon are often used in polymer flame retardant systems for their synergistic effect [27]. However, single flame retardants usually have high contents in order to achieve an excellent flame-retardant effect, which is not good for the conductivity.

**Figure 1:**
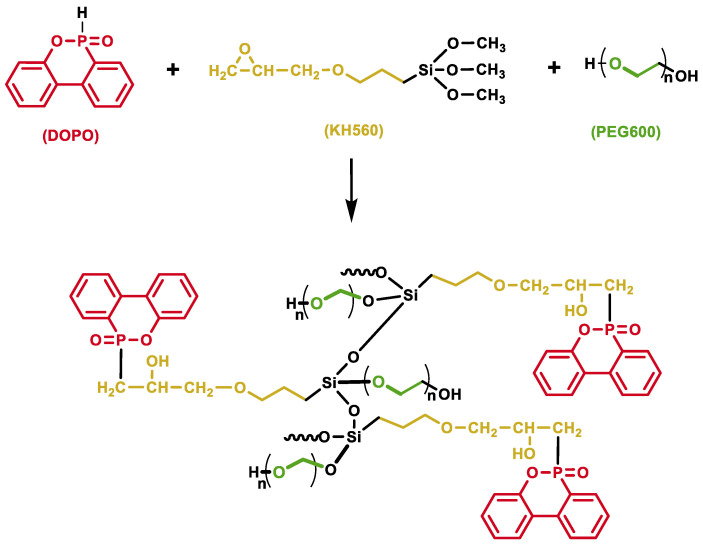
Synthesis of DKPpolymer.

In this paper, phosphorus-containing DOPO and silicon-containing KH560 monomers were introduced into PEG600 (DKP) chain to synergistically improve the flame resistance, and the ratio of PEG-based electrolyte to lithium salt was adjusted to obtain an optimized electrochemical performance. KH560 has typical epoxy groups and methoxy groups which can react with the active P–H bonds of DOPO, and the abundant hydroxyl groups on PEG600 can easily polymerize with the methoxy group of KH560 [28]. DOPO and KH560 were copolymerized with PEG600 to produce a novel intrinsic flame-retardant polymer electrolyte, as shown in Figure 1, which improves the compatibility between additives and matrix. The introduced chemical bond improved the stability of the matrix so as to obtain a better flame-retardant performance.

## 2. Experiment

### 2.1. Materials

Polyethylene glycol 600 (PEG600), 3-glycidoxy-propyltrimethoxysilane (KH560), 9,10-dihydro-9-oxa-10-phosphaphe-nanthrene-10-oxide (DOPO), lithium bis(trifluoromethane sulfonate) (LiTFSI) and anhydrous acetonitrile (CH_3_CN) were purchased from Aladdin (St. Louis, MO, USA).

### 2.2. Samples Preparation

#### 2.2.1. Preparation of DOPO-KH560-PEG600 Polymer

Nineteen grams of PEG600 and 5 g KH560 were added into a 500 mL three-necked flask. The flask was magnetically stirred and heated to 160 °C under argon atmosphere and then heated for 10 h with the addition of 3.8 g DOPO. The product was distilled under reduced pressure to remove the unreacted small molecules, and the viscous polymer DOPO-KH560-PEG600 (DKP) was obtained.

#### 2.2.2. Preparation of DOPO-KH560-PEG600/LiTFSI Polymer Solid Electrolyte

LiTFSI and DKP polymer were dissolved in anhydrous acetonitrile according to the mass ratios of 8:1, 10:1, 12:1, 20:1 and 30:1. The mixture was then stirred magnetically for 12 h. The fully mixed solution was coated on a tetrafluoroethylene mold and dried at 60 °C for 24 h to obtain the DOPO-KH560-PEG600/LiTFSI (DKP/LiTFSI) solid electrolyte. The DKP/LiTFSI solid electrolytes with different mass of LiTFSI are named as DKP/LiTFSI-8, DKP/LiTFSI-10, DKP/LiTFSI-12, DKP/LiTFSI-20 and DKP/LiTFSI-30.

#### 2.2.3. Preparation of LiFePO_4_|DOPO-KH560-PEG600/LiTFSI|Li Battery

##### The Preparation of LiFePO_4_ Cathode

The active material LiFePO_4_ powder, binder polyvinylidene fluoride (PVDF) and conductive carbon black (CB) were mixed at a ratio of 8:1:1, and *N*-methyl-2-pyrrolidinone (NMP) was added to form a uniform slurry. The slurry was coated on the surface of aluminum foil and then dried in a vacuum oven at 100 °C for 24 h. The LiFePO_4_ cathode was prepared by punching the coated Al foil into disks with 13 mm diameter.

##### The Preparation of Button Lithium Ion Battery

The CR2032 batteries were assembled with a lithium sheet as the negative electrode, LiFePO_4_ as the positive electrode and DKP/LiTFSI membrane as the electrolyte. The assembly of LIBs was completed in a glove box filled with argon.

### 2.3. Experimental Measurements

Infrared spectroscopy (FT-IR) was carried out using a Nicolet apparatus (Thermo Scientific, Waltham, MA, USA). Nuclear magnetic resonance spectrometer (NMR) was carried out on AV III from Bruker Company (Billerica, MA, USA). Gel permeation chromatography (GPC) was tested on Waters 1525 with tetrahydrofuran as the mobile phase. Thermogravimetric analysis (TG) was tested under nitrogen atmosphere with thermogravimetric analyzer (TG209F1, NETCHI, Selbu, Germany) in the temperature range from 30 to 800 °C and a heating rate of 10 °C/min. Scanning electron microscopy (SEM, Manufacturer, OR, USA) of FEI (Quanta 250) with the accelerating voltage of 20 kV showed the morphology of samples. The heat release rate was tested by a microcalorimeter (MCC) from Govmark company with the temperature ranging from 70 to 750 °C at a heating rate of 1 °C/s.

The AC impedance analyzer (302N, Autolab, Herisau, Switzerland) was used to characterize the conductivity of electrolyte membranes in the frequency range from 10^−1^ to 10^6^ Hz. The bulk resistance (R_b_) of solid polymer electrolytes was obtained by AC impedance spectroscopy, and the conductivity of electrolytes was calculated according to the Equation (1).

*σ* = *L*/(*R* × *S*) (1)

where *L* is the thickness of solid electrolyte membrane, *R* is the bulk resistance of the solid electrolyte membrane and *S* is the area of the solid electrolyte membrane [29]. The electrochemical stability window of electrolytes was tested by the linear sweep voltammetry (LSV) method with VMP3 (Bio-Logic Company, Paris, France), with a stainless steel sheet used as a working electrode and lithium sheets as the counter electrode and reference electrode. CR2032 was then tested for galvanostatic charge/discharge with a Wuhan Landian CT2001A tester (Wuhan, China).

## 3. Results and Discussion

### 3.1. Characterization of the Structure and Thermal Properties of DKP Polymer

**Figure 2:**
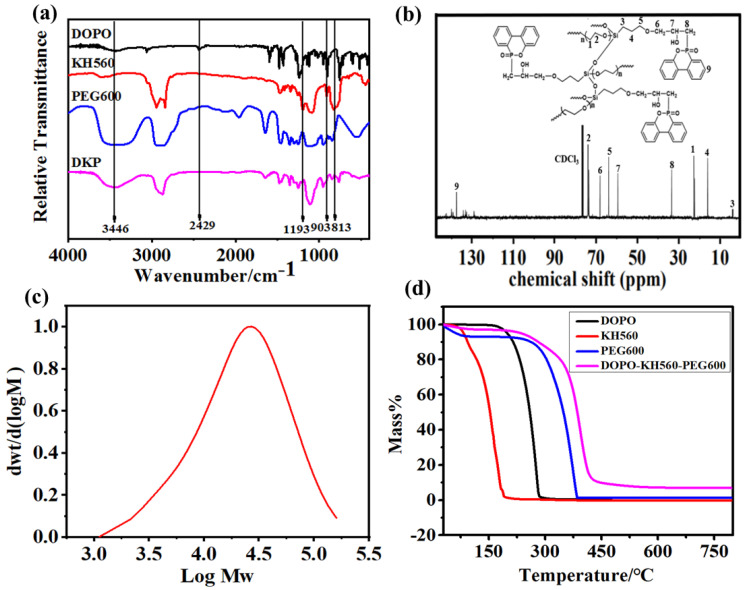
(**a**) FT-IR spectra of DOPO, KH560, PEG600 and DKP polymer; ^13^C NMR spectrum (**b**) and GPC curve (**c**) of DKP polymer; (**d**) TG curves of DOPO, KH560, PEG600 and DKP polymer.

The FTIR spectra of KH560, DOPO, PEG600 and DKP polymer are shown in Figure 2a, respectively, where the absorption peak at 2429 cm^−1^ presents the characteristic peak of P–H bond in DOPO [30], and the absorption peak at 1193cm^−1^ is the typical peak of Si–O–CH_3_ in KH560. Two peaks at 903 and 813 cm^−1^ reveal the expansion and contraction vibration of epoxy groups in KH560 [31], and the absorption peak at 3466 cm^−1^ is the characteristic absorption peak of –OH group in PEG600 [32]. It is noticed that the absorption peaks of P–H bond at 2429 cm^−1^ and Si–O–CH_3_ at 1193 cm^−1^ both disappeared in the DKP polymer because of the ring-open reaction between DOPO and KH560. In addition, the intensities of typical epoxy groups and –OH are obviously decreased, suggesting that the active reaction was performed between PEG-600 and KH560. The above results show that the synthesis has been implemented according to our design.

The ^13^C NMR spectrum of DKP polymer is shown in Figure 2b. The multiple peaks between 130 and 140 ppm correspond to the resonance absorption peaks of biphenyl on DKP polymer. The peak at 34.7 ppm is the resonance absorption of carbon atoms in P–CH_2_ [33], and two peaks at 63.5 and 68.2 ppm correspond to two kinds of carbon atoms in –CH_2_–O–CH_2_– of KH560 [30]. The peaks at 22.7 and 73.2 ppm reflect the two types of carbon atoms in –CH_2_–CH_2_–O– of PEG600 part in the DKP polymer. GPC results (Figure 2c) show there is only one weight average molecular weight (Mw) distribution around 33,193, suggesting that the PEG-600 molecules have been removed under the reduced-pressure process. The NMR results are consistent with the FTIR results, both indicating that DKP was successfully synthesized.

The thermal decomposition temperatures were revealed from the TG curves (Figure 2d), where KH560, DOPO and PEG-600 delivered thermal decomposition temperatures of 91.5, 195.7 and 273.1 °C, respectively, and the residue amounts were all close to 0 wt%. For the DKP, its thermal decomposition temperature and residue rate increased to 303 °C and 10.9 wt%, respectively, indicating that the cross-linked structure can improve the thermal stability of the DKP polymer. Meanwhile, the Si and P elements can play a synergistic flame retardant role, where the P atoms can promote the formation of a carbon layer [34] while Si atoms stabilize the generated carbon layer [28], resulting in jointly promoting the thermal stability and residue rate of DKP polymer.

### 3.2. Combustion Analysis of DKP/LiTFSI Solid Electrolyte

**Figure 3:**
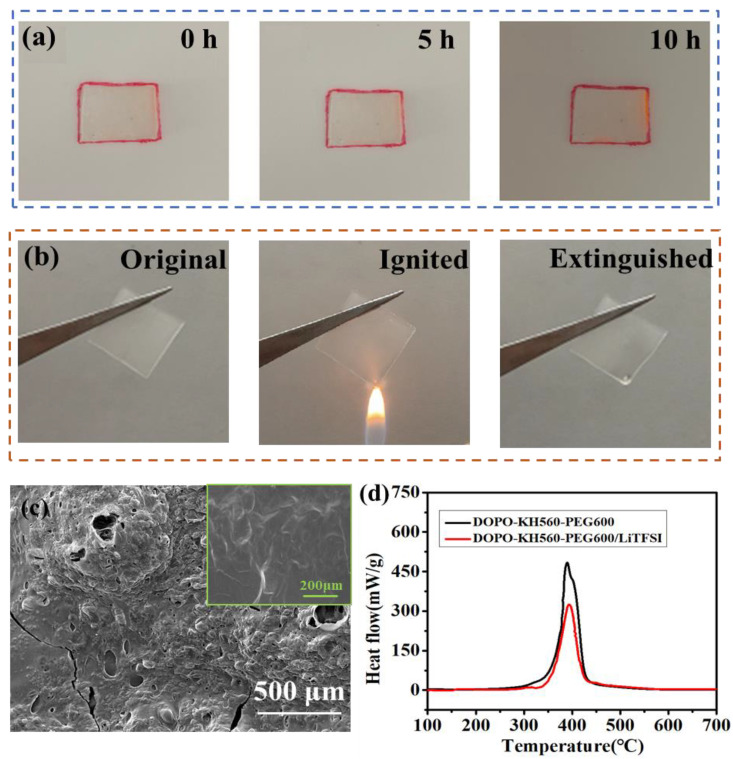
(**a**) Dimensional stability and (**b**) self-extinguishing property of DKP/LiTFSI solid electrolytes before and after being ignited; (**c**) SEM images of DKP/LiTFSI electrolyte after igniting, inset: before igniting; (**d**) HRR curves of DKP polymer and DKP/LiTFSI solid electrolytes.

Figure 3a shows the dimensional stability of DKP/LiTFSI solid electrolyte membrane at 60 for 0, 5 and 10 h. There was no change in size of the electrolyte membrane after bearing at 60 °C after 10 h, indicating the excellent thermal stability of the electrolyte membrane. A combustion test of DKP/LiTFSI electrolyte membrane in air is shown in Figure 3b. It is noticed that the solid electrolyte membrane did not ignite when set on fire; it just melted by the high temperature of the flame, showing excellent flame retarding ability [35]. The SEM image (Figure 3c inset) shows the uniform and dense morphology of an electrolyte membrane with no obvious lithium salt particles. A dense residue layer was generated after burning, as shown in Figure 3c, which benefits the prevention of further combustion of DKP/LiTFSI electrolytes. HRR curves can describe the heat released by the combustion of materials in unit time. The HRR curves of DKP and DKP/LiTFSI solid electrolyte membranes are displayed in Figure 3d, where the DKP polymer showed the maximum heat release rate at 390 °C, while the electrolyte membrane compounded with LiTFSI had the maximum heat release rate at 395 °C. Such a high heat release temperature is beneficial to restrain the thermal runaway of the battery.

### 3.3. Electrochemical Performance of DKP/LiTFSI Solid Electrolyte

**Figure 4:**
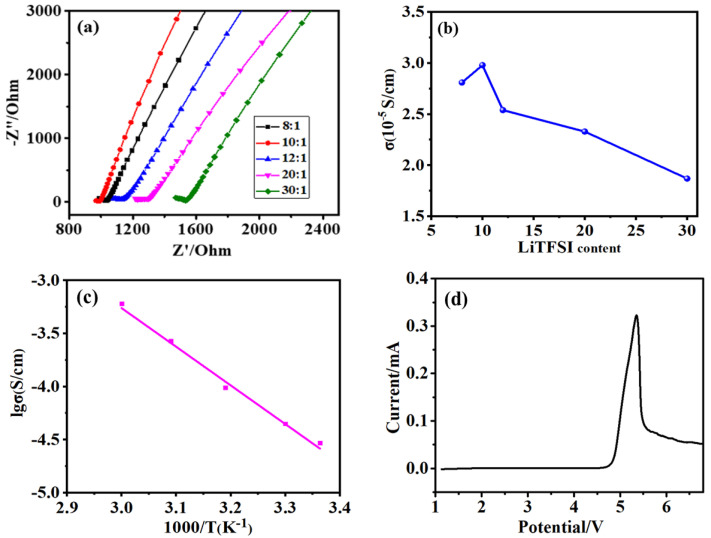
(**a**) AC impedance spectrum and (**b**) conductivity of DKP-based solid electrolytes with different LiTFSI contents; (**c**) the temperature dependence curves of ionic conductivity of DKP/LiTFSI electrolytes; (**d**) anodic oxidation curve of DKP/LiTFSI electrolytes.

The AC impedance spectrograms and the conductivity of DKP/LiTFSI electrolytes with different lithium salt contents are shown in Figure 4a,b. Since the DKP polymer has a Si–O center with low rotation barrier, which is beneficial to chain segment movement, the DKP/LiTFSI electrolyte shows relatively high ionic conductivity [22]. As shown in Figure 4a, the conductivity of lithium ion first increased and then decreased with the increase in the lithium salt content. The conductivity reached the highest value of 2.98 × 10^−5^ S/cm at 25 °C with a mass ratio of 10:1 (LiTFSI:DKP). This is because the conductivity of electrolyte membranes is mainly determined by the number of free lithium ions and the mobility of lithium ions. With the increase in the amount of lithium salt, the free lithium ions in the electrolyte gradually increased, resulting in an increase in the effective carrier and ion conductivity. However, lithium salt cannot be dissociated effectively, and ions are associated in pairs with a high content of lithium salt. The conductivity of the electrolyte will be finally weakened following the increased viscosity and decreased mobility.

An Arrhenius plot for the ionic conductivity of DKP/LiTFSI-10 as a function of temperature is depicted in Figure 4c, where the conductivity of the electrolyte was tested at 25, 30, 40, 50 and 60 °C. At 25 °C, the obtained ionic conductivity was 2.98 × 10^−5^ S·cm^−1^, and its ionic conductivity was as high as 5.96 × 10^−4^ S·cm^−1^ when the temperature rose to 60 °C. The conductivity increased with the increasing temperature and corresponds well with the Arrhenius equation (Equation (2)):

(2) $$\sigma =\sigma_{0}exp^{\left(-Ea/kT\right)}$$

where *σ_0_* is the ionic conductivity of solid electrolytes at 0 K, *T* corresponds to the test temperature, *k* presents the Boltzmann constant, and *Ea* is the activation energy (*Ea*). Furthermore, the electrochemical stability of DKP/LiTFSI-10 working at 25 °C was investigated by an LSV test. As shown in Figure 4d, there was no obvious oxidation taking place up to 4.8 V versus Li^+^/Li, which makes it difficult for the reaction between electrolytes and electrodes.

**Figure 5:**
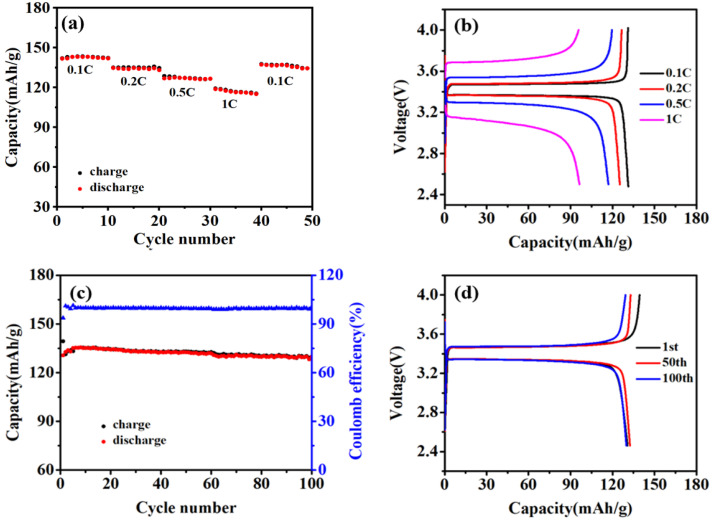
(**a**) Rate performance; (**b**) charge–discharge curves at different current densities; (**c**) cyclic performance and (**d**) charge–discharge curves at 0.2 C of LiFePO_4_|DKP/LiTFSI|Li all-solid-state batteries at 60 °C.

In order to evaluate the electrochemical performance of the battery at high temperatures, the rate and cycling performance of a LiFePO_4_/Li all-solid-state battery with the DKP/LiTFSI-10 solid electrolytes were further tested at 60 °C. Figure 5a,b illustrates the rate performance and charge–discharge curves at the current densities of 0.1 C (1 C = 170 mA/g), 0.2, 0.5 and 1 C; the corresponding discharge specific capacities after cycling for 10 cycles were 142.0, 133.2, 126.6 and 115.5 mAh/g, respectively. The all-solid-state battery delivered a specific capacity of 134.5 mAh/g when the current density was reset to 0.1 C. What is more, Figure 5b reveals that the difference of the charging and discharging platforms was only 0.12 V at 0.1 C, indicating a lower polarization of the cell endured at high temperature. The low polarization at high temperature means the redox reaction of the cell was efficient, and the internal resistance at the electrode/electrolyte interface also weakened the cell’s overpotential. Meanwhile, the Coulombic efficiency was almost close to 100%. Figure 5c,d exhibits the cycling performance and charge–discharge curves of the LiFePO_4_/Li all-solid-state battery at 0.2 C. The battery delivered a charge and discharge capacity of 139.4 and 130.7 mAh/g, with a coulombic efficiency of 93.7%, for the first cycle. After six cycles, its reversible capacity gradually remained stable, and the coulombic efficiency was close to 100% during the long cycling. These results indicate that the LiFePO_4_/Li all-solid-state battery assembled with DKP/LiTFSI electrolytes can be well applied and that they exhibit excellent cycling and rate performance at a high temperature.

At the same time, some parameters are compared in Table 1. The LiFePO_4_/Li all-solid-state battery possessed high capacity, had capacity attenuation within the acceptable range and also had high ionic conductivity. In this work, we discussed the electrochemical performance of the battery at higher temperatures, and the electrochemical performance of the battery at low temperatures was not involved. Since most lithium batteries are used at the temperature of about 25–40 °C [36], the battery performance at low temperatures may be studied in our future work.

## 4. Conclusions

In this paper, a novel solid polymer electrolyte with high conductivity that can be used stably at high temperatures was prepared by the chemical grafting of PEG. The synergistic flame-retardant effect was realized by introducing the phosphorus to promote the formation of a carbon layer and silicon atoms to further stabilize the generated carbon layer, which gives the LiFePO_4_/Li all-solid-state battery excellent electrochemical performance at high temperatures. In addition, the battery showed superior rate performance at high conductivity (5.96 × 10^−4^ S·cm^−1^) and an efficient redox reaction at 60 ℃. Therefore, the synthesized synergistic flame-retardant polymer is a promising electrolyte and shows application potential in solid-state lithium-ion batteries.

## Figures and Tables

**Table 1:** The comparison of PEO-based SPEs performance for the application in a lithium battery.

Cell	Capacity (mAh/g)	Temp (°C)	Ionic Conductivity (S/cm)	Ref
Cellulose-PEO-PCA-LiBOB (LiFePO_4_/Li)	150 at 0.07 C 138 at 2 C	60	1.3 × 10^−5^	[37]
P(STFSILi)-PEO-P(STFSILi) (LiFePO_4_/Li)	153 at 0.1 C 118 at 1 C	60	3 × 10^−4^	[38]
PEO/DPMB-LiTFSI (LiFePO_4_/Li)	150 at 0.02 C	55	4.7 × 10^−5^	[39]
BDM-PDG-LiTFSI (LiFePO_4_/Li)	140 at 0.1 C 35 at 1 C	70	6.5 × 10^−4^	[40]
cellulose/PEG LiTFSI (LiFePO_4_/Li)	153.3 at 0.2 C 97 at 1 C	55	6.1 × 10^−3^	[41]
DKP-LiTFSI (LiFePO_4_/Li)	142.0 at 0.1 C 115.5 at 1 C	60	5.96 × 10^−4^	This work
